# Deep Research Agents: Major Breakthrough or Incremental Progress for Medical AI?

**DOI:** 10.2196/88195

**Published:** 2026-03-26

**Authors:** Matthew Yu Heng Wong, Ariel Yuhan Ong, David A Merle, Pearse A Keane

**Affiliations:** 1 School of Clinical Medicine, University of Cambridge, Addenbrooke's Hospital, Hills Rd, Cambridge, CB2 0SP, United Kingdom, 44 07888263149; 2 NIHR Biomedical Research Centre, Moorfields Eye Hospital NHS Foundation Trust, London, United Kingdom; 3 Institute of Ophthalmology, University College London, London, United Kingdom

**Keywords:** artificial intelligence, AI, medical research, scientific writing, large language models, LLMs

## Abstract

Deep research agents are autonomous large language model–based systems capable of iterative web search, retrieval, and synthesis. They are increasingly positioned as the next major leap in medical artificial intelligence. In this viewpoint, we argue that while these agents mark progress in information access and workflow automation, they represent an incremental evolution rather than a paradigm shift. We review current applications of deep research agents in biomedical scenarios, including literature review generation, clinical evidence synthesis, guideline comparison, and patient education. Across these early use cases, the tools demonstrate the ability to rapidly gather and structure up-to-date information, often producing outputs that appear comprehensive and well-referenced. However, these strengths coexist with unresolved and clinically significant limitations. Citation fidelity remains inconsistent across models, with subtle misinterpretations or unreliable references still common. Their retrieval processes and evidence-ranking mechanisms remain opaque, raising concerns about reproducibility and hidden biases. Moreover, overreliance on artificial intelligence–generated syntheses risks eroding clinicians’ critical appraisal skills and may introduce automation bias at a time when medicine increasingly requires deeper scrutiny of information sources. Safety constraints are also less predictable within multistep research pipelines, increasing the risk of harmful or inappropriate outputs. Finally, current evidence is largely limited to proof-of-concept evaluations, with little evidence from real-life clinical deployment. We contend that deep research agents should be embraced as assistive research tools rather than pseudoexperts. Their value lies in accelerating information gathering, not replacing rigorous human judgment. Realizing their potential will require transparent retrieval architectures, robust benchmarking, and explicit educational integration to preserve clinicians’ evaluative reasoning. Used judiciously, these systems could enrich medical research and practice; used uncritically, they risk amplifying errors at scale. We contend that deep research agents should be embraced as assistive research tools rather than pseudoexperts. Their value lies in accelerating information gathering, not replacing rigorous human judgment. Realizing their potential will require transparent retrieval architectures, robust benchmarking, and explicit educational integration to preserve clinicians’ evaluative reasoning. Used judiciously, these systems could enrich medical research and practice; used uncritically, they risk amplifying errors at scale.

## Introduction

Large language models (LLMs) have now become extensively tested and used for a range of medical tasks and capabilities [1,2]. However, current LLMs are limited by issues such as hallucinations, opaque reasoning processes, and reliance on prior static training data. Retrieval-augmented generation (RAG) aims to mitigate some of these limitations by allowing LLMs to draw from external information sources (eg, specialized medical textbooks and journals) [3]. In late 2024, another advancement in the development of LLMs was introduced in the form of “deep research agents”—autonomous research assistants that combine LLMs with real-time internet search and citation capabilities [4]. These agents leverage the capabilities of RAG by replacing this external data source with up-to-date, live internet access [5].

Beyond simple responses to standard chat-based prompting, these deep research agents can autonomously perform web searches, retrieve, cross-reference, and cite literature, positioning themselves as potential replacements for a myriad of human-led tasks. OpenAI’s Deep Research (launched in February 2025) was designed to rapidly access and analyze vast datasets of literature, synthesize their findings, and then generate tailored written outputs [6]. These tools promise to bridge the gap between an ever-growing medical knowledge base and the limited capacity clinicians have to digest it. With minimal user input, they can autonomously generate comprehensive, well-structured, and referenced reviews of prespecified topics. In this viewpoint, we use “deep research agents” (lowercase) to denote the general class of agentic, multistep, research-oriented LLM systems. The capitalized term “Deep Research” is reserved exclusively for specific commercial implementations (eg, OpenAI’s Deep Research) and is used only where product-level distinctions are necessary.

Deep research agents are a relatively recent development, and their application in biomedical contexts remains only partially investigated. In this study, we review how deep research agents have been explored in early use cases in medicine, ranging from literature reviews and medical education to clinical queries, highlighting their anticipated potential. We also identify areas where these agents are limited and require further study and careful validation as usage and adoption grow. Overall, we argue that while these agents mark progress in information access and workflow automation, they represent an incremental evolution in medical artificial intelligence (AI) rather than a paradigm shift.

## Deep Research Agents Versus Conventional LLM Chatbots for Medicine

**Figure 1.**
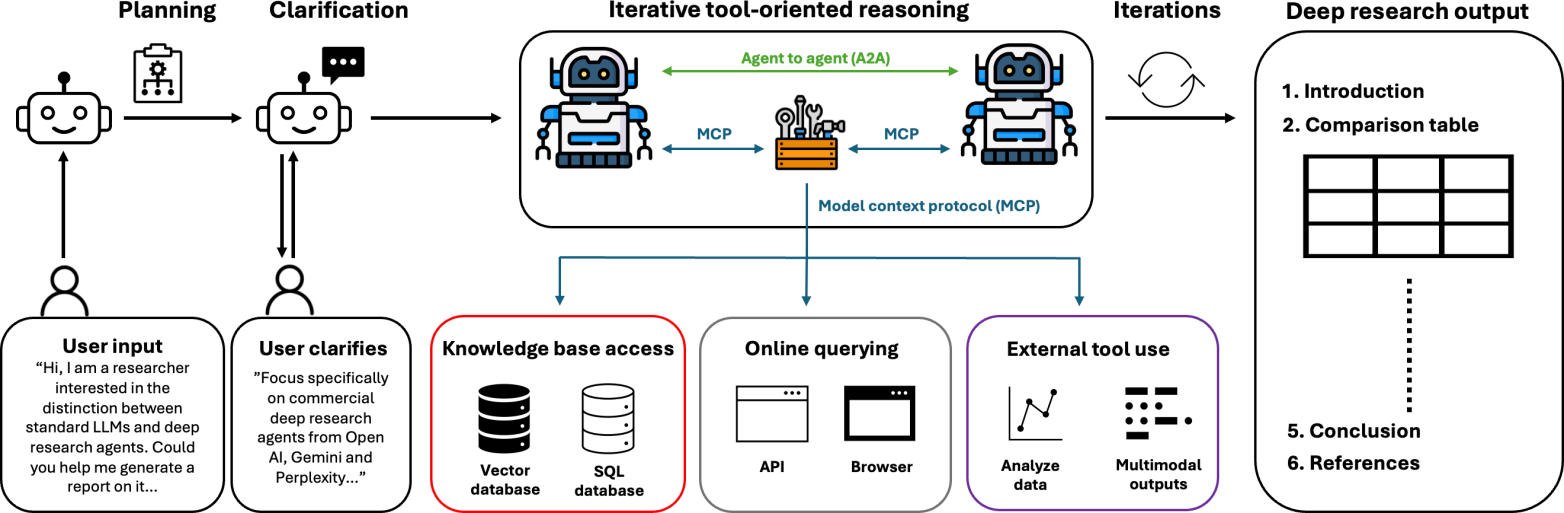
Deep research agents autonomously guide the entire workflow: starting from user input, optionally clarifying intent and planning the search, iteratively using tools for offline data retrieval (databases, vector stores), online search (APIs, browsers), and higher-level functions such as analytics or multimodal content creation (text, image), before assembling the findings into a structured report [8]. API: application programming interface; LLM: large language model.

Deep research agents represent a new evolution of conventional chatbot-style LLMs, which integrate RAG workflows and more complex attention mechanisms. Unlike standard LLM chatbots, which typically rely on the model’s internal weights and a single input context window, deep research systems typically embed a multistep reasoning pipeline: they first query external knowledge sources, retrieve relevant documents, and then feed those passages into the generator, which uses cross-attention to integrate information across multiple sources before producing a response (Figure 1). Importantly, unlike single-shot retrieval in traditional RAG pipelines, web exploration and source retrieval are iterative and agent-driven, enabling deeper coverage of sparse or scattered evidence [7].

Deep research agents extend RAG by combining retrieval with real-time tool use and iterative reasoning, allowing the system not only to fetch relevant documents but also to plan, refine, and synthesize information as part of a single workflow. Dynamically engaging with external tools and managing multistage research tasks in real time ostensibly allows these agents to provide greater autonomy, context-awareness, and accuracy in their outputs (Table 1) [8].

**Table 1.** Architectural and functional comparison of deep research agents vs standard retrieval-augmented generation chatbots.

Dimension	Deep research agents (eg, OpenAI Deep Research, Gemini Deep Research, Perplexity Pro)	Standard RAG^a^ or web-enabled Chatbots (single-shot retrieval + generation; optional browsing)
Core behavior	Multistep autonomous pipeline: *plan → search → retrieve → read → synthesize → cite*. Persistent reasoning and external grounding.	Nonagentic workflow: answer generation supported by one-shot retrieval or ad hoc browsing; limited planning and iteration.
External information integration	Integrated web and file retrieval; dynamic multisource querying with internal memory.	Single-pass retrieval (*top-k* docs); weaker systematic coverage and cross-source reconciliation.
Processing time or run length	Long-running sessions (≈5‐30 min typical) processing many documents and verifying citations.	Short latency (seconds to a few minutes) typically fewer tool calls and shallower document review.
Citation handling or attribution	Structured outputs with traceable references and in-line links; still requires human verification for claim-source alignment.	Can provide citations when configured or prompted. May show weaker claim-source alignment.
RAG	Full multihop RAG with iterative query refinement, retrieval, cross-checking, and synthesis.	Single-hop RAG; limited multihop behavior unless manually orchestrated.
Control and reasoning architecture	Agentic controller layer atop the LLM^b^ for planning, tool selection, and multisource integration.	Fixed LLM + retriever. Reasoning and evidence integration occur implicitly within a single LLM pass.
Output style	Structured research reports with citations and sectioning (eg, “Findings,” “Limitations”), can be tailored with prompt engineering.	Conversational prose or short summaries without specific prompting.
Limitations or risks	Improved grounding but still susceptible to retrieval errors, misweighting of evidence, opaque rankings of sources, and hallucinations.	Similar risks (retrieval noise, cherry-picked snippets, hallucinations) and weaker systematic coverage. Higher risk of hallucinated references.
Potential use cases	Systematic reviews and evidence synthesis, where provenance and depth matter, and slow generation is acceptable.	Point queries, quick updates, short summaries with citations. For tasks where speed matters.

a RAG: retrieval-augmented generation.

b LLM: large language model.

Compared with their early 2025 iterations, current deep research agents have shifted from text-only, single-model research bots into multimodal, tool-integrated, multimodel research systems. This evolution includes enhanced perceptual capabilities, allowing agents to interpret not only text but also visual elements such as figures, images, and complex documents, alongside expanded access to public sources (eg, PDFs from the web) through external connectors. In parallel, successive model upgrades have incorporated advanced self-reflection mechanisms, increases in scalability, and longer context windows. For example, newer iterations of Gemini’s Deep Research employ “Flash Thinking” to support more explicit planning and iterative self-critique during synthesis [9].

**Figure 2.**
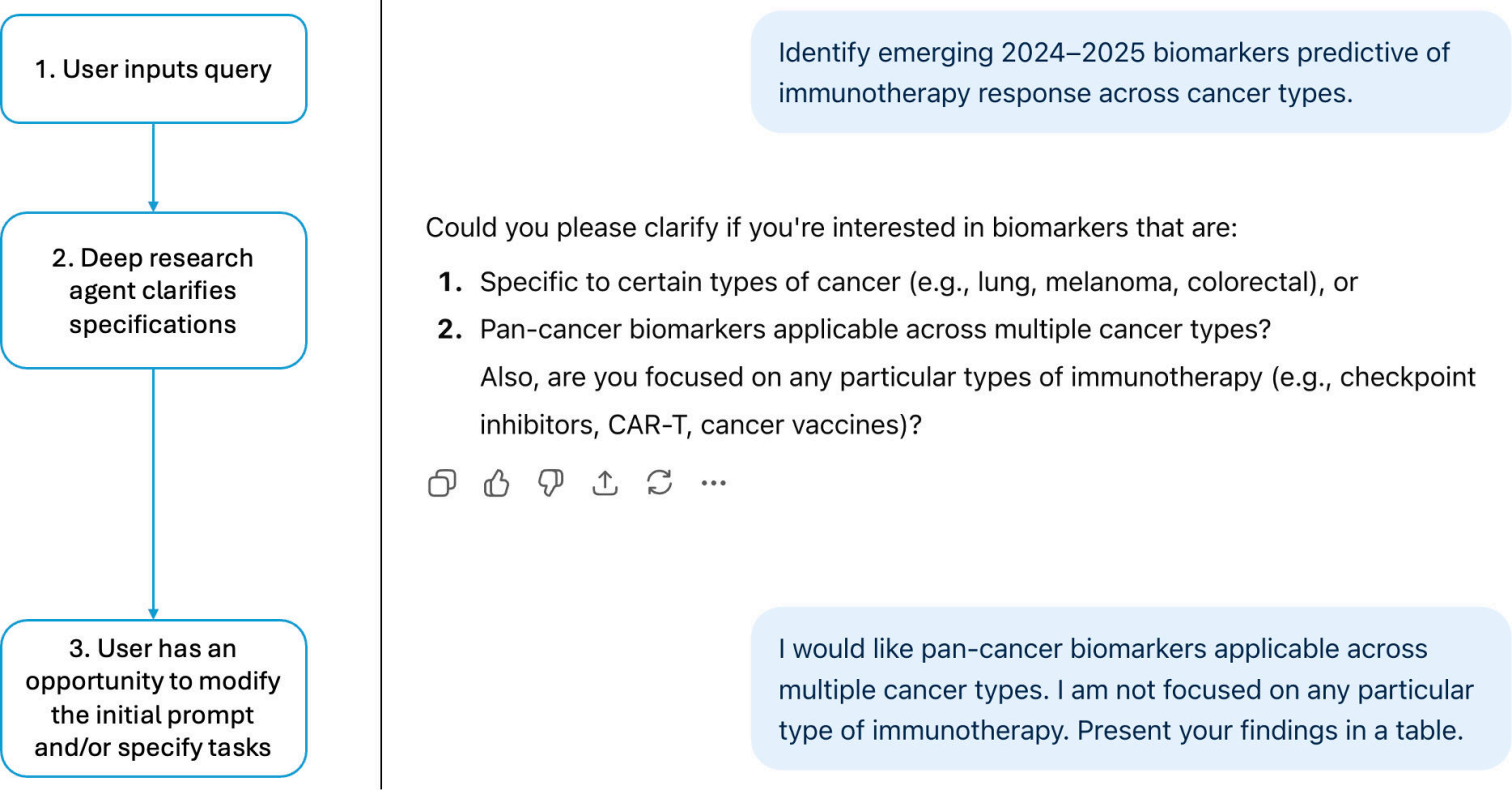
An example of how a deep research agent (OpenAI’s Deep Research) engages in an iterative dialog with the user to clarify intent before conducting literature searches. By confirming details such as cancer type and biomarker scope, the agent supports a more focused and structured synthesis—a workflow that extends beyond typical single-turn large language model interactions.

From a clinical perspective, this architecture could one day transform how evidence is synthesized and applied in medicine and health care. By autonomously retrieving and cross-referencing peer-reviewed studies, clinical guidelines, and trial reports, deep research agents could assist in rapid reviews**,** clinical decision support, and precision medicine literature surveillance (Table 2). For instance, a clinician could enter a query to “Identify emerging 2024‐2025 biomarkers predictive of immunotherapy response across cancer types” and receive a sourced, citation-backed synthesis reflecting the latest evidence—something standard LLM chatbots, limited to static parametric memory, cannot reliably provide. This output strongly hints at the future of AI-assisted knowledge retrieval. Moreover, the use of cross-attention between retrieved documents allows the model to contextualize and reconcile conflicting studies**,** potentially supporting meta-analytic or guideline-development workflows [8]. We briefly tested this workflow and evaluated the resulting retrieval and output of the model (Figures2  3).

**Table 2.** Illustrative deep research queries showcasing the breadth of use cases for clinicians and academics.

Category	Example query	Potential benefits
Clinical updates	“What emerging biomarkers beyond programmed death-ligand 1 predict response to immunotherapy, based on recent clinical studies?”	Provides clinicians with current knowledge without manually searching journals and reviewing multiple articles.
Guideline comparison	“How do European Society for Medical Oncology and American Society of Clinical Oncology guidelines address immunotherapy response in lung cancer?”	Rapid evidence synthesis across guideline bodies.
Basic science research	“What biological mechanisms underlie emerging biomarkers predictive of immunotherapy response?”	Elucidates underlying molecular mechanisms and pathways.
Translational research	“Which emerging biomarkers are sufficiently validated and assay-ready to inform immunotherapy patient selection?”	Highlights cutting-edge translational trends.
Patient communication	“Explain in simple terms to a patient how their biomarkers may help predict whether immunotherapy will work.”	Generates layperson-friendly patient information.
Academic writing	“Draft an introduction for a review summarizing emerging biomarkers predictive of immunotherapy response.”	Saves time on first-draft writing.
Whimsical or exploratory	“What will clinicians recognize as the moment when immunotherapy response first became meaningfully predictable?”	Engages creativity and unlocks novel perspectives.

**Figure 3.**
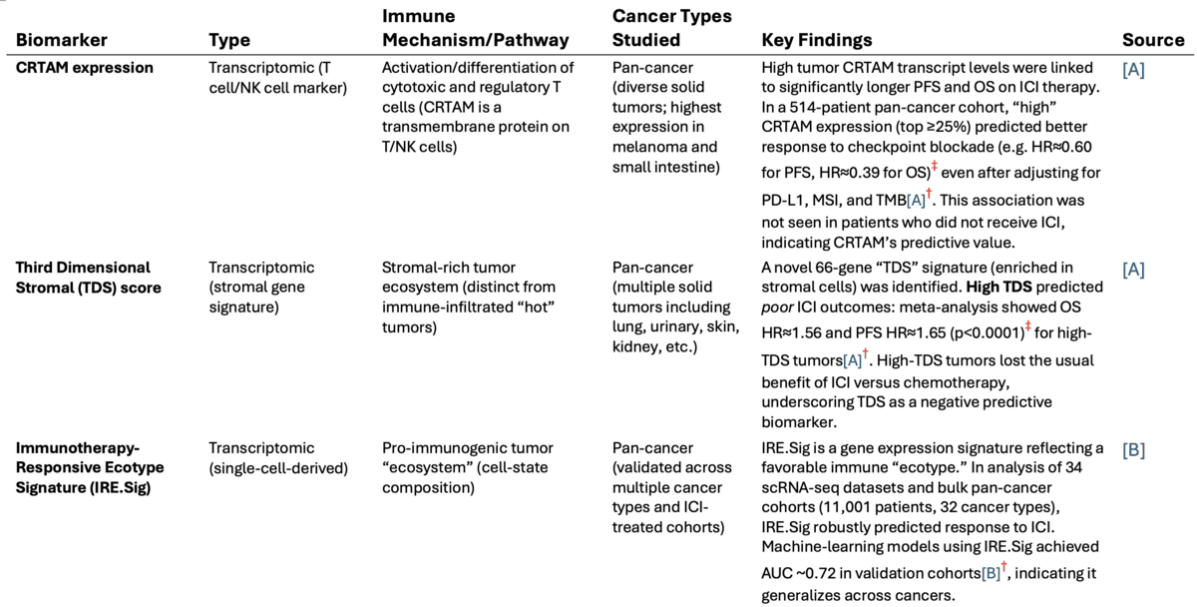
Example of a structured evidence table generated by a deep research agent in response to the earlier query, “identify emerging 2024‐2025 biomarkers predictive of immunotherapy response across cancer types.” The agent autonomously retrieved, synthesized, and formatted recent literature into a reference-supported summary in less than 8 minutes. In this instance, all Deep Research–generated content and citations were independently checked against the original publications and were found to accurately reflect the source literature, with no hallucinated references. This figure, therefore, illustrates a successful deep research workflow. However, polished outputs of this kind should not be assumed to be uniform across tasks or systems. The superscript annotations highlight illustrative areas where known risks may arise: ^†^potential inconsistencies in citation fidelity (eg, incorrect or semantically mismatched source attribution) and ^‡^possible overstatement or imprecise reporting of quantitative effects. These annotations are hypothetical and do not imply errors in the example shown but are included to emphasize the need for verification despite apparent surface reliability. Citations in this figure are shown in Multimedia Appendix 1. AUC: area under the curve; CTRAM: class I–restricted T-cell–associated molecule; ICI: immune checkpoint inhibitor; MSI: microsatellite instability; OS: overall survival; PD-L1: programmed death-ligand 1; PFS: progression-free survival; TMB: tumor mutational burden.

Importantly, although multihop retrieval and iterative self-correction introduce greater autonomy and coverage, the underlying architecture of these agents remains conceptually continuous with established RAG techniques. Their principal innovation lies in the orchestration layer that coordinates planning, retrieval, and tool use, rather than in a fundamentally new reasoning paradigm (eg, no symbolic reasoning or formal logic introduced). These agents may therefore represent a stepwise functional improvement in autonomy but are only an incremental evolution in architecture.

As such, deep research agents inherit many of the same fundamental limitations as prior RAG-based systems. Current implementations remain limited by their dependence on open-access data, lack of transparent ranking mechanisms, and inability to independently verify information. Consequently, these systems continue to operate as unpredictable black boxes, generating probabilistic responses with limited reasoning transparency. As these constraints are progressively addressed, deep research tools may increasingly contribute to the early stages of systematic reviews and evidence-based clinical support.

## Deep Research Agents for Literature Reviews and Knowledge Synthesis

One potential use of deep research agents is drafting medical literature reviews or summaries of evidence. Ong et al [5] evaluated several such tools (Gemini 1.5 Pro Deep Research, Perplexity’s Deep Research, and OpenAI’s Deep Research) by tasking them with writing a literature review on “oculomics” (ocular biomarkers of systemic health). They found that the OpenAI agent produced the most compelling draft—a logically structured, readable, and informative narrative that nonexpert readers could hardly distinguish from a human-written review. In another study, Jesudason et al [10] reported that OpenAI’s Deep Research could generate a multipage referenced text on complex medical topics in minutes, including controversial clinical questions such as antithrombotic therapy for carotid dissection. Meanwhile, Wang et al’s [11] DeepEvidence allows deep research to be performed across various biomedical knowledge graphs, achieving substantial performance gains in evidence synthesis across drug discovery and clinical trial development. Such comprehensive reports, complete with citations, suggest deep research agents could save researchers substantial time on initial literature reviews.

Another study benchmarked multiple agents for systematic review title and abstract screening in radiology. They found traditional LLMs showed variable sensitivity but high specificity in classifying abstracts, while deep research agents (OpenAI and Google’s Gemini) could autonomously retrieve literature with high precision but moderate recall [12]. Another recent evaluation by Loke et al [13] benchmarked deep research agents (OpenAI, Perplexity, and Gemini) and autonomous agentic systems like Manus AI against a published systematic review and meta-analysis on myopia risk. Although this work shares partial authorship with the present manuscript, it suggests systems such as OpenAI and Perplexity Deep Research can identify studies meeting inclusion criteria and perform generally accurate qualitative data extraction, indicating utility for certain stages of the review process.

However, these studies also reveal important caveats. Citation accuracy arises as a common concern. Ong et al [5] reported multiple instances of hallucinations or erroneous citations, including references to nonexistent articles and incorrect attribution of fictitious articles to real researchers. In independent tests, Keplinger et al [14] tasked 5 deep research agent models with generating a dermatology literature review. They found OpenAI’s Deep Research performed best in reference accuracy—approximately 95% of its citations were identifiable, with approximately 70% completely correct. In contrast, Google Gemini 2.5 Pro Deep Research and Perplexity AI Deep Research frequently produced fabricated citations: roughly 47% to 50% of references from those models had fake authors or titles. Another study similarly found OpenAI’s Deep Research and xAI’s Grok 3 achieved citation precision exceeding 90%, surpassing Gemini and Perplexity but still clearly lagging behind human reviewers [15]. Together, these findings show that while deep research agents can rapidly synthesize literature, citation reliability remains inconsistent across models and still falls short of human standards.

Citation–claim alignment is another notable problem across the board. Keplinger et al [14] found that over 50% of OpenAI’s Deep Research’s cited statements contained at least one subtle inaccuracy or misrepresentation of the source. The AI often misinterpreted study results, cited out of context, or hallucinated details not in the cited papers. These citation errors are difficult to detect without thorough cross-checking by an expert, raising concerns that an AI-generated review could unwittingly propagate misinformation. Ong et al [5] similarly observed factual errors in the AI-generated oculomics review that appeared innocuous to nonexperts—for example, claims about nonexistent trials or incorrect statements about regulatory approvals. Meanwhile, Loke et al [13] found that none of the evaluated deep research agents could conduct a genuine meta-analysis or generate accurate, publication-quality figures and tables. For instance, frequent omissions and hallucinations were observed when agents were asked to construct summary tables of included studies.

Besides accuracy, bias represents a key concern. Deep research agents often rely on RAG-modeled retrieval to select what they consider the “best” or most relevant evidence, using vector similarity search, ranking algorithms, and other techniques. However, the opacity of what the model deems to be “optimal” or “most relevant” presents a real challenge to the generation of verifiable, reproducible knowledge in clinical and translational research [16]. Given that English-language publications from Western nations dominate the internet, deep research tools may be more likely to draw from these, which risks reinforcing global inequalities in biomedical research. Without conscious efforts to diversify their input sources, these agents risk perpetuating existing publication biases and overlooking unconventional or minority perspectives in research [17].

In summary, deep research agents can dramatically speed up information gathering or evidence summarizing in medicine, producing text that is well organized and richly referenced. However, the consensus among all studies is that human oversight remains crucial for now.

## Deep Research Agents for Patient Education and Clinical Q&A

Another emerging medical application of deep research agents is in patient education and answering clinical questions. By integrating up-to-date information from the web, these agents can potentially serve as advanced, real-time medical chatbots. Gültekin et al [18] compared OpenAI’s Deep Research with DeepSeek R1 (a contemporary model with a similar “deep think” web-search capability) for answering common patient questions about anterior cruciate ligament surgery. Notably, OpenAI’s Deep Research delivered more comprehensive answers, covering questions in greater depth (completeness score 4.0 vs 3.2 for DeepSeek). DeepSeek’s answers, however, were clearer and more accessible to laypeople, written at a significantly lower reading level (8.9 vs 14.2). The authors noted that excessive detail may overwhelm patients, while oversimplification risks omitting critical information. This suggests integrating the strengths of both models (the thoroughness of ChatGPT and the plain-language clarity of DeepSeek) could be ideal for patient education materials. Nevertheless, readability need not be an intrinsic model limitation: deep research systems can ultimately be prompted to adjust language complexity, allowing clinicians to tailor explanations to patient literacy levels.

A similar multimodel evaluation by Yetkin et al [19] queried 11 AI chatbots—including GPT-4 Deep Research, Perplexity’s Deep Research mode, and others—with common patient questions about sarcoidosis. The models that integrated RAG (ChatGPT-4.0 Deep Research, Perplexity, and xAI’s Grok Deep Search) achieved the highest scores on validated metrics such as DISCERN (measuring the quality of health publications) and Patient Education Materials Assessment Tool (PEMAT; patient education and understandability). These deep research–enabled models significantly outperformed standard chatbots that lacked web access. However, the improved comprehensiveness came again at the expense of readability. The top-performing models delivered information at a very high reading level—Flesch-Kincaid grade more than 16 (college-level difficulty). This reiterates the importance of thoughtful prompting and clinician oversight to align outputs with patient health literacy.

Taken together, these studies show that deep research agents can dramatically accelerate the generation of high-quality patient education content, but their output may not yet be patient-accessible without clinical mediation. In practice, a clinician could use deep research agents to quickly gather accurate medical information for a patient, then refine the output into more digestible terms. Moreover, patients have become increasingly AI literate and can adopt simple prompting strategies (eg, “rewrite this explanation for an eighth-grade reading level” or “explain this using analogies and avoid medical jargon”) to request clearer, lay-friendly reformulations of AI-generated content. However, while prompting can improve readability, it cannot guarantee accuracy. Patients need to be made aware of the potential pitfalls of AI-generated medical information, including the importance of corroborating outputs against trusted sources (eg, National Health Service patient information resources). As time progresses, deep research agents could gradually shift the clinician’s role from information provider to information steward: verifying accuracy, simplifying complexity, and ensuring alignment with a patient’s health literacy level and emotional needs.

## Emerging Medical-Specific Deep Research Agents and Benchmarks

As deep research agents have matured, evaluation has shifted from general retrieval toward complex, multistep reasoning in medical contexts. Medically tailored benchmarks have been introduced alongside the development of domain-specific deep research agents.

For example, MedBrowseComp was introduced in 2025 as the first benchmark for autonomous, multihop biomedical research [20]. It featured over 1000 physician-curated questions that simulate realistic clinical research tasks. Importantly, MedBrowseComp evaluated agents not solely on output accuracy but on their ability to locate evidence across multiple authoritative sources and reconcile fragmented biomedical information. The benchmark revealed that leading proprietary deep research agents, including Open AI’s o3, Gemini 2.5 Pro, and Perplexity, could answer only around a quarter of these multistep questions correctly.

Meanwhile, domain-specialized agents such as IQVIA’s Med-R1 (an 8B parameter medical reasoning model with an agentic framework) and the open-source MedResearcher-R1 (32B) emerged. These incorporated dense medical knowledge and custom retrieval tools, enabling them to match and even outperform much larger general models on certain biomedical tasks. On MedBrowseComp, MedResearcher-R1 (32B) achieved a new state-of-the-art 27.5% accuracy, surpassing OpenAI’s o3 Deep Research (25.5%) and Google’s Gemini 2.5 Pro (~25%) [21]. Likewise, IQVIA’s Med-R1 Deep Research Agent achieved comparable results to OpenAI’s o3 and Google’s Gemini 2.5 Pro despite using only an 8B parameter model [22]. These medical-specific systems demonstrate more advanced multihop clinical reasoning and synthesis across specialized databases (eg, PubMed, clinical trial registries, and Food and Drug Administration data). By leveraging domain-specific retrieval engines and expert knowledge, they may be able to synthesize complex chains of biomedical facts that generalist agents may miss. In addition, these specialized deep research agents attain their results with relatively compact model sizes (8B for IQVIA or 32B parameters for MedResearcher-R1). This efficiency suggests promising potential for deployment in computationally constrained health care settings, where smaller, domain-focused AI systems may be integrated.

Current limitations underscore that adapting these techniques to medical contexts is still in its early stages. Tasks requiring beyond 4 to 5 reasoning hops remain largely unsolved, as even state-of-the-art agents saw a sharp drop in accuracy on 5-hop questions. Their performance profile on these benchmarks reinforces that such systems should not yet be considered reliable clinical assistants, but rather exploratory tools requiring substantial human oversight. Moreover, the scope of the MedBrowseComp benchmark is itself constrained: it uses automated answer judging (without full clinical expert review) and focuses on hematology and oncology datasets alone, omitting certain subdomains such as advanced genomics or multimodal cases.

## Benefits and Limitations of Current Deep Research Agents

Current studies illustrate an emerging picture of the potential roles of deep research agents in biomedical settings. In summary, these agents are being explored as advanced research assistants and information mediators: to draft literature reviews, summarize evidence on controversies, assist in writing academic papers, educate patients with up-to-date information, and even support clinical decision queries. The common thread is their ability to autonomously comb through and synthesize vast text corpora to produce a structured, referenced answer in a fraction of the time a human might take.

This has already led some to herald a new era of AI-augmented medical research. Editorials have described “AI’s deep research revolution” and the “next evolutionary leap” [23], noting that current iterations of LLMs can speed up the summarization of literature and even facilitate comparing study results—such that “for text-based research, such as systematic reviews or meta-analyzes, AI may replace them efficiently” [15]. Scientific publishing may be entering a “post-LLM” era, where AI agents equipped with deep reasoning and retrieval capabilities are reshaping how literature is synthesized and reviewed.

**Figure 4.**
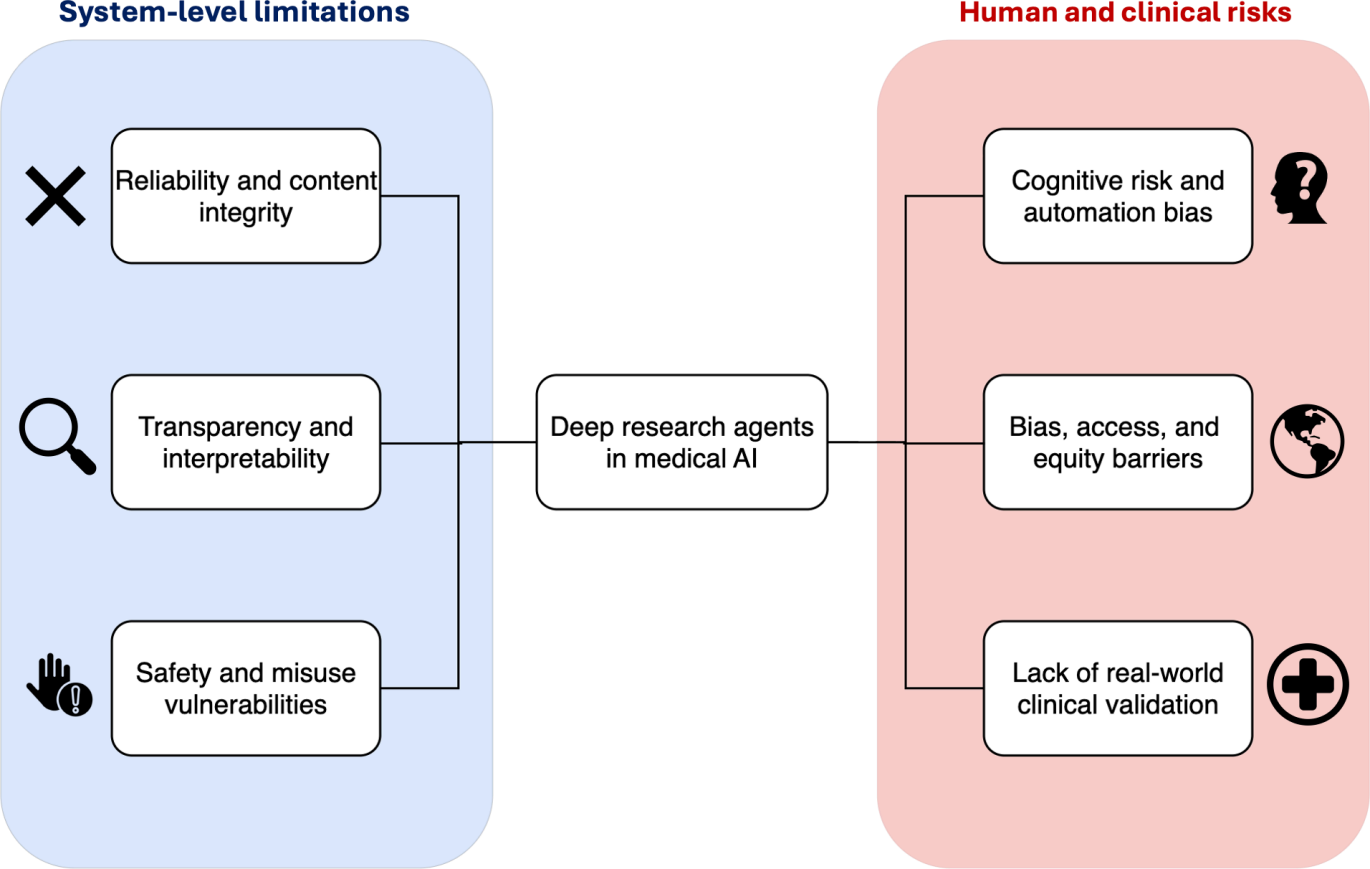
Limitations for deep research agents include system-level reliability and safety, human and societal risks, and the lack of real-world clinical validation. AI: artificial intelligence.

On the other hand, virtually all medical evaluations of these tools so far have included strong warnings (Figure 4). A recurring concern is the integrity of citations and content. While the latest models have largely solved the problem of blatantly fabricated references that plagued earlier LLMs, subtler issues persist—slight errors in bibliographic details or correct references attached to unsupported claims. These are not always obvious, especially to nonexperts or when an AI output looks polished. If clinicians or researchers take AI-generated text at face value, there is a risk of false information seeping into publications or clinical guidance.

Another concern is the potential for overreliance. Jesudason et al [10] urge that medical trainees should avoid leaning too heavily on AI-prepared syntheses, as they may lose the ability to critically appraise evidence themselves. Cognitive off-loading, where learners delegate lower-level mental tasks to external systems to free up working memory, can support higher-order reasoning and enhance learning if used judiciously [24]. By contrast, automation bias—an overreliance on automated systems for important tasks—arises when users defer to system outputs even when independent judgment is required. In medical training, critical appraisal of studies, evidence weighting, data interpretation, and clinical reasoning represent non-negotiable competencies that should not be off-loaded. Excessive off-loading of these tasks risks fostering not only automation bias (overreliance on automated systems) but also deskilling (loss of previously acquired competencies), never-skilling (failure to develop essential expertise), and mis-skilling (reinforcement of incorrect reasoning due to AI errors or bias) [25]. Over time, excessive reliance on predigested syntheses may erode clinicians’ capacity for deep critical thinking, analogous to early concerns about the erosion of manual skills with surgical robotics. In the same vein, Keplinger et al [14] expressed concerns that outsourcing the initial draft of a review to AI could distort authors’ own mental map of the field and undermine their engagement with the literature, ultimately harming scientific creativity.

Another key but often overlooked aspect is safety. Recent work has raised broader concerns that increasing autonomy and multistep planning in AI systems introduces qualitatively new safety challenges, particularly around oversight and loss of control [26]. In addition, it has been suggested that deep research agents may circumvent traditional safety filters under certain conditions. Unlike standalone LLMs, which are engineered to refuse unsafe prompts, deep research agents’ multistep reasoning pipelines may potentially weaken those refusal mechanisms. Chen et al [27] note that deep research agents may process a broader range of query types than standard chat interfaces because the research pipeline is treated as an information-gathering task rather than a direct response to a prompt. This could inadvertently allow malicious or high-risk instructions—such as instructions related to biological weapons development, hacking, or privacy breaches—to be pursued under the guise of research. This does not mean safety filters are obsolete or permanently bypassed in research-oriented systems. Rather, they highlight a current tension between autonomous information-gathering workflows and content moderation mechanisms that were originally designed for short “chatbot” conversational use. As vendors continue to iterate on agentic architectures and develop guardrails specific to these agents, such safety vulnerabilities may be mitigated.

Importantly, ethical and practical limitations exist. Deep research agents are constrained to open-access information sources, as they cannot directly retrieve paywalled journal content, which could bias them toward certain sources. Their outputs also reflect the data they find—if the online content is skewed or outdated, the AI’s output will inevitably be biased as well. Moreover, access to deep research agents themselves is likely to be uneven. Owing to their computational intensity, such systems are expected to be deployed through subscription-based or premium access models. This introduces an economic barrier that may limit adoption in resource-constrained settings, potentially reinforcing disparities in global medical research. As a result, agentic research workflows may not only reflect prevailing publication biases but also exacerbate inequities in who is able to perform large-scale, synthesis-driven research. Over time, wider licensing or open-source alternatives may mitigate these effects, but economic access remains an important consideration in the near term. Moreover, the development of smaller, lightweight models with deep research capabilities (eg, IQVIA’s Med-R1 8B model) may enable more computationally affordable evidence synthesis in resource-limited settings.

Transparency is another issue: it is not always clear how AI algorithms prioritize which sources to use or what reasoning led to a given conclusion. This opacity can make it hard for users to fully trust the results without verification, especially in medicine, where decisions require provenance and auditability. This is in contrast to evidence retrieval platforms such as OpenEvidence, which demonstrate a more transparent paradigm. OpenEvidence pulls directly from curated biomedical databases (eg, PubMed/Medline, clinical trial registries) and exposes every citation used, allowing users to trace each claim back to the original paper or paragraph [28,29]. In the future, deep research systems could adopt a similar architecture—combining autonomous reasoning with constrained, citation-verifiable retrieval pipelines.

Finally, there remains a notable lack of studies that rigorously evaluate the real-world capabilities of deep research agents within medicine. The existing literature largely consists of perspective pieces and proof-of-concept studies, with few empirical studies providing systematic benchmarking of these agents against established evidence synthesis standards or real-world clinical deployment. This gap underscores the need for methodologically robust research to determine whether deep research agents can reliably replicate or augment real-world medical workflows [30]. Recent efforts have begun to recognize this benchmarking gap. Du et al [31] introduced *DeepResearchBench*, the first comprehensive, multidomain benchmark specifically designed for deep research agents. Other initiatives, such as Rigorous Bench, further highlight how current evaluation schemes for LLMs fail to capture the multistep reasoning, citation integrity, and evidence synthesis capabilities characteristic of deep research systems [32]. Together, these studies emphasize that robust, clinically tailored benchmarks are urgently needed to measure not only the generative fluency of these agents but also their fidelity to source material, transparency of reasoning, and reproducibility of evidence synthesis. Moving forward, such benchmarks should also incorporate clinically relevant outcomes, including time saved, workload reduction, and decision support quality.

## A Practical Verification and Transparency Framework for Clinical Use

To support the judicious use of deep research agents in clinical and research settings, we propose a structured evidence verification and transparency framework operating at both the system level and the end-user level.

At the system level, developers and vendors should adopt standards for logging retrieval queries, source rankings, and cross-attention or contribution weights linking generated statements to retrieved passages. While these signals do not provide full interpretability of model reasoning, they could enable partial auditing of multistep workflows by exposing which documents were retrieved and which sources most strongly influenced each synthesized claim [33]. Importantly, these logs should be made requestable and interpretable for clinicians and researchers, recognizing that domain experts (rather than system developers) are best positioned to interpret biomedical relevance and evidence quality. As these signals are not currently exposed by most proprietary systems, they should be regarded as forward-looking policy and design recommendations for model developers rather than tools currently available for clinical end-users.

At the user level, clinicians can follow a three-stage workflow when assessing agent outputs: (1) checking that citations originate from authoritative clinical repositories such as PubMed, Food and Drug Administration, guideline bodies, etc; (2) conducting selective audits of the most clinically actionable statements against original papers or guidelines, ensuring these claims are backed by multiple independent sources; and (3) assessing semantic coherence and task alignment to confirm the output remains focused on the specified clinical scope and does not exhibit internal contradictions or overconfidence in weak evidence. By combining architectural transparency with verification at the clinician level, this framework may structure responsible evaluation and improve confidence in the adoption of deep research agents for biomedical evidence synthesis.

Ultimately, trade-offs between the speed advantages of deep research agents and the time required for rigorous manual verification are likely, particularly in high-stakes clinical use. In practice, the net time savings of these systems may arise less from end-to-end automation than from accelerating early-stage information gathering and narrowing the search space for subsequent human verification.

## Conclusion

The evolution of general-purpose LLMs to more specialized research-oriented agents, including deep research agents (eg, Open AI’s Deep Research, Google’s Gemini Deep Research, and Perplexity’s Deep Research), marks significant progress in how medical knowledge can be accessed and synthesized. However, while they may represent a stepwise functional improvement in workflow automation, they remain an incremental extension of existing reasoning architectures and inherit many of their limitations. Current evaluations highlight the need for careful, evidence-based integration into medical practice. Their outputs continue to require human scrutiny to ensure fidelity, as they can miscite, misrepresent, or overgeneralize evidence in ways that are not immediately obvious. In medicine, these tools should complement, but not replace, rigorous human-driven research and review processes.

From a practical perspective, the idea of a “deep research” revolution may already be here, and if used judiciously, it can empower health care professionals—speeding up literature analysis and freeing up human experts to focus on critical thinking, creative insights, and patient-centered decision-making. We believe the most sustainable path forward lies in collaboration with these agents, grounded in transparency and clinical accountability.

## Supplementary material

10.2196/88195 Multimedia Appendix 1 Raw chat logs with Open AI’s Deep Research.

## Abbreviations

AI: artificial intelligence
LLM: large language model
PEMAT: Patient Education Materials Assessment Tool
RAG: retrieval-augmented generation
