# Supplementary material for: Deep Research Agents: Major Breakthrough or Incremental Progress for Medical AI?
Source: J Med Internet Res. 2026 Mar 26;28:e88195. doi: 10.2196/88195 (PMC13021100; doi:10.2196/88195)
Supplement: Multimedia Appendix 1 [file jmir-v28-e88195-s001.docx]

**Section 1: Chat-log with ChatGPT’s Deep Research**


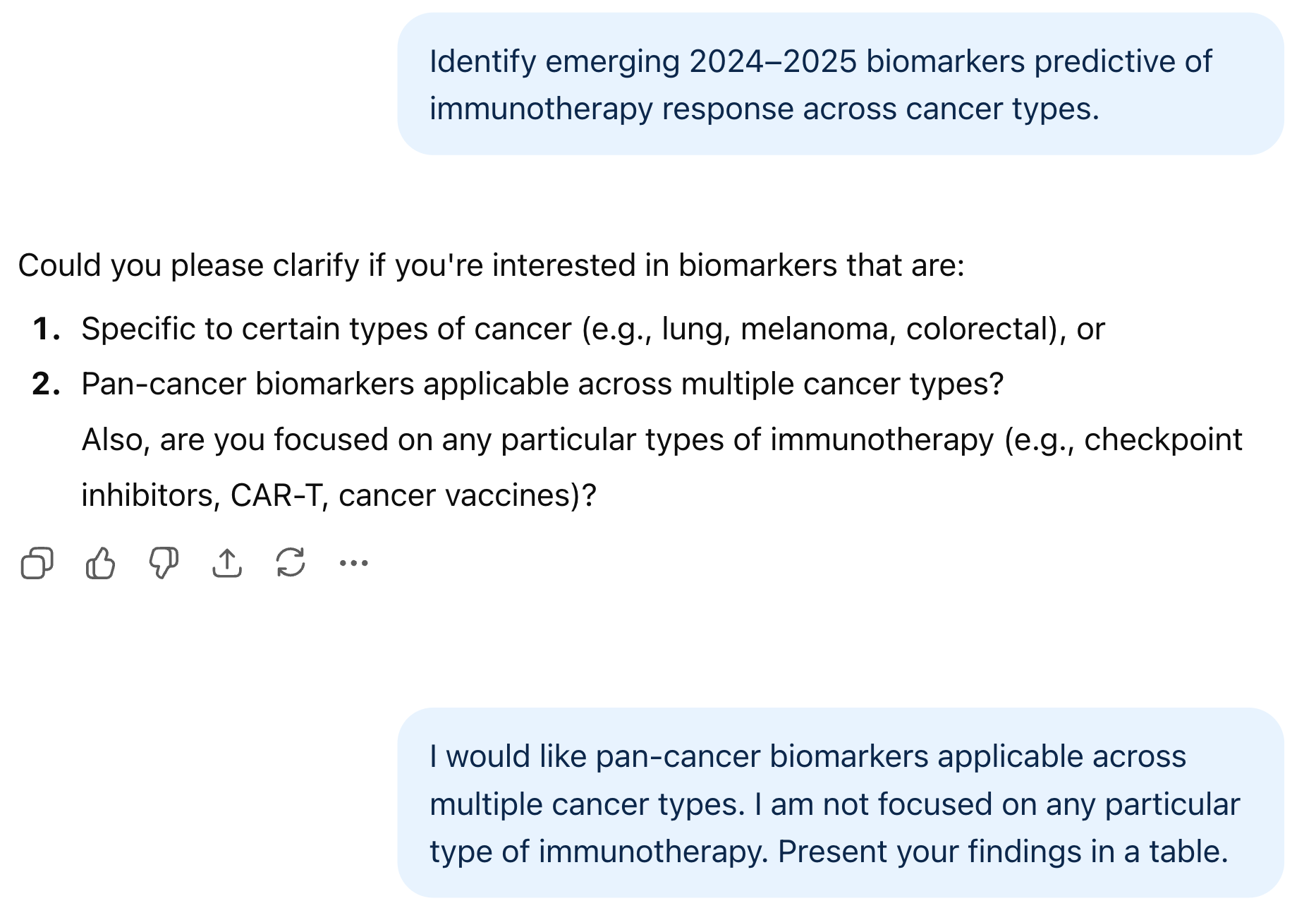


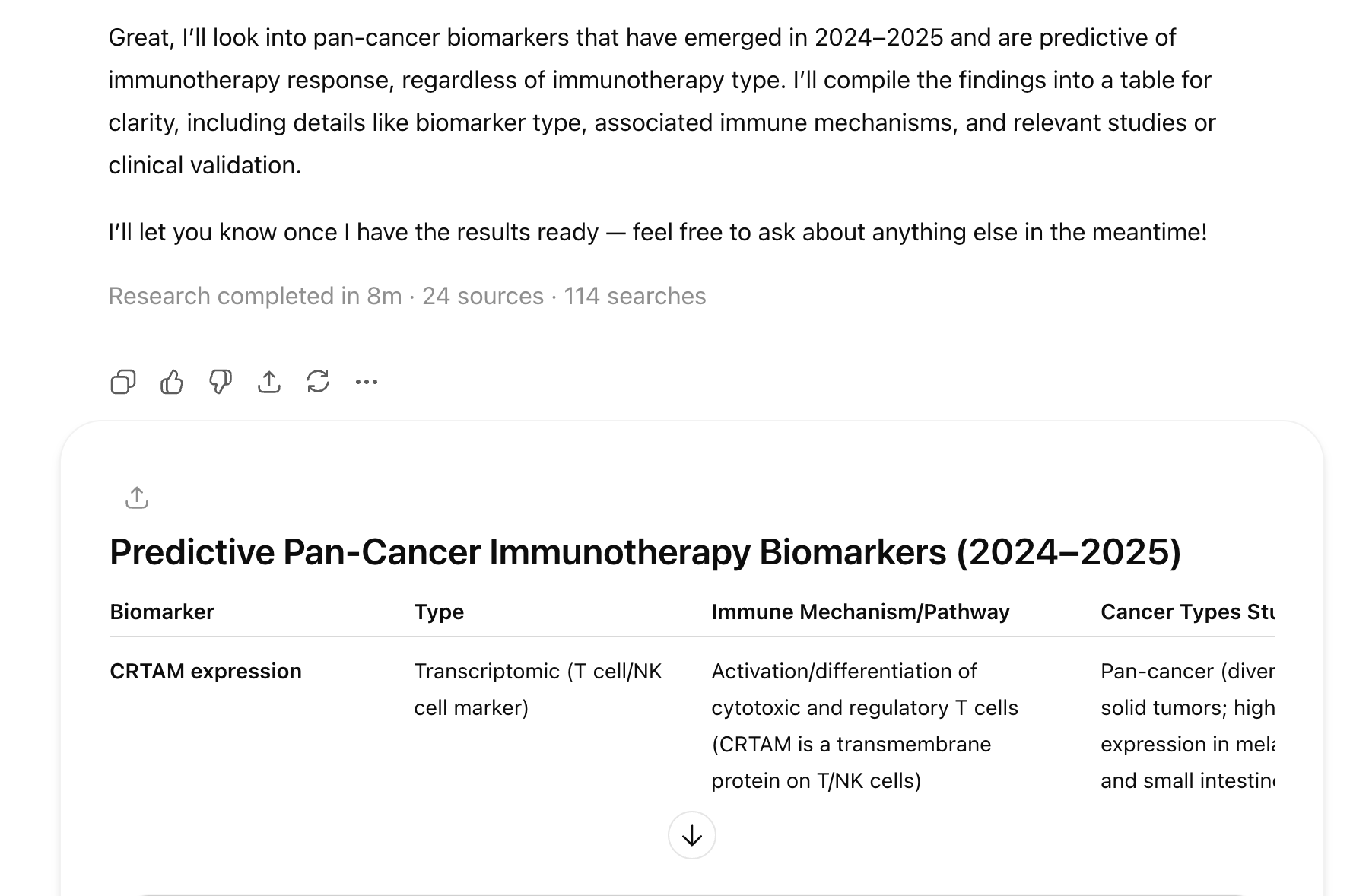


Full (unedited) Deep Research Output and references:

# Predictive Pan-Cancer Immunotherapy Biomarkers (2024–2025)

| **Biomarker** | **Type** | **Immune Mechanism/Pathway** | **Cancer Types Studied** | **Key Findings** | **Source** |
| --- | --- | --- | --- | --- | --- |
| **CRTAM expression** | Transcriptomic (T cell/NK cell marker) | Activation/differentiation of cytotoxic and regulatory T cells (CRTAM is a transmembrane protein on T/NK cells) | Pan-cancer (diverse solid tumors; highest expression in melanoma and small intestine) | High tumor CRTAM transcript levels were linked to significantly longer PFS and OS on ICI therapy. In a 514-patient pan-cancer cohort, “high” CRTAM expression (top ≥25%) predicted better response to checkpoint blockade (e.g. HR≈0.60 for PFS, HR≈0.39 for OS) even after adjusting for PD-L1, MSI, and TMB[[1]](https://s3.amazonaws.com/files.oncologymeetings.org/prod/s3fs-public/2024-05/AM24-Developmental-Therapeutics-Immunotherapy.pdf#:~:text=showed%20high%20CRTAM%20expression,significance%20was%20preserved%20in%20multivariate). This association was not seen in patients who did not receive ICI, indicating CRTAM’s predictive value. | [[1]](https://s3.amazonaws.com/files.oncologymeetings.org/prod/s3fs-public/2024-05/AM24-Developmental-Therapeutics-Immunotherapy.pdf#:~:text=showed%20high%20CRTAM%20expression,significance%20was%20preserved%20in%20multivariate) |
| **Third Dimensional Stromal (TDS) score** | Transcriptomic (stromal gene signature) | Stromal-rich tumor ecosystem (distinct from immune-infiltrated “hot” tumors) | Pan-cancer (multiple solid tumors including lung, urinary, skin, kidney, etc.) | A novel 66-gene “TDS” signature (enriched in stromal cells) was identified. **High TDS** predicted *poor* ICI outcomes: meta-analysis showed OS HR≈1.56 and PFS HR≈1.65 (p<0.0001) for high-TDS tumors[[2]](https://s3.amazonaws.com/files.oncologymeetings.org/prod/s3fs-public/2024-05/AM24-Developmental-Therapeutics-Immunotherapy.pdf#:~:text=its%20enrichment%20in%20subsets%20of,over%20Chemo%20were%20lost%20in). High-TDS tumors lost the usual benefit of ICI versus chemotherapy, underscoring TDS as a negative predictive biomarker. | [[2]](https://s3.amazonaws.com/files.oncologymeetings.org/prod/s3fs-public/2024-05/AM24-Developmental-Therapeutics-Immunotherapy.pdf#:~:text=its%20enrichment%20in%20subsets%20of,over%20Chemo%20were%20lost%20in) |
| **Immunotherapy-Responsive Ecotype Signature (IRE.Sig)** | Transcriptomic (single-cell-derived) | Pro-immunogenic tumor “ecosystem” (cell-state composition) | Pan-cancer (validated across multiple cancer types and ICI-treated cohorts) | IRE.Sig is a gene expression signature reflecting a favorable immune “ecotype.” In analysis of 34 scRNA-seq datasets and bulk pan-cancer cohorts (11,001 patients, 32 cancer types), IRE.Sig robustly predicted response to ICI. Machine-learning models using IRE.Sig achieved AUC ~0.72 in validation cohorts[[3]](https://www.nature.com/articles/s41698-024-00703-w?error=cookies_not_supported&code=da9116d5-b174-41ca-87f8-4dc79eebfa0c#:~:text=we%20utilized%20EcoTyper%20and%20publicly,machine%20learning%20models%20successfully%20predicted), indicating it generalizes across cancers. | [[3]](https://www.nature.com/articles/s41698-024-00703-w?error=cookies_not_supported&code=da9116d5-b174-41ca-87f8-4dc79eebfa0c#:~:text=we%20utilized%20EcoTyper%20and%20publicly,machine%20learning%20models%20successfully%20predicted) |
| **ENLIGHT matching score (EMS-NGS)** | Transcriptomic (AI-derived algorithm) | Integrated tumor-immune expression profile | Pan-cancer (ICB-treated patients across ~14 solid tumor types) | ENLIGHT is an AI algorithm that computes a matching score from tumor RNA-seq. In pan-cancer trials, EMS-NGS significantly outperformed conventional biomarkers: responders had higher EMS-NGS pre-treatment (median ~0.92 vs 0.62 in non-responders, p<0.0002). EMS-NGS achieved AUC ~0.74 for ICI response (vs 0.70 for PD-L1 IHC, 0.64 for TMB)[[4]](https://www.researchgate.net/publication/392302319_Validation_of_ENLIGHT_an_AI_predictor_of_immune_checkpoint_blockade_ICB_response_and_resistance_across_the_treatment_span#:~:text=vs%200,6). Its predictive score remained concordant through treatment and could be derived from standard H&E slides (as EMS-DP). | [[4]](https://www.researchgate.net/publication/392302319_Validation_of_ENLIGHT_an_AI_predictor_of_immune_checkpoint_blockade_ICB_response_and_resistance_across_the_treatment_span#:~:text=vs%200,6) |
| **cfDNA methylation & fragmentation** *(CSM/FLS via cfMeDIP-seq)* | Epigenomic (circulating tumor DNA) | Tumor-specific DNA methylation and cfDNA fragment-length changes | Pan-cancer (mixed solid tumors on pembrolizumab) | A “tumor-naïve” cfDNA assay (cfMeDIP-seq) was used to track early changes in patients on pembrolizumab. By computing a cancer-specific methylation (CSM) score and fragment-length score (FLS) from plasma, researchers found that *early changes* in these metrics predicted clinical benefit and survival[[5]](https://pmc.ncbi.nlm.nih.gov/articles/PMC11145176/#:~:text=demonstrate%20that%20early%20changes%20in,in%20patients%20treated%20with%20pembrolizumab). In other words, this blood-based methylome/fragmentome signature provided a noninvasive predictive biomarker of ICI response across diverse cancers. | [[5]](https://pmc.ncbi.nlm.nih.gov/articles/PMC11145176/#:~:text=demonstrate%20that%20early%20changes%20in,in%20patients%20treated%20with%20pembrolizumab) |
| **Gut microbiome enterotypes / Phenylacetylglutamine (PAGln)** | Microbiome/metabolomic | Microbiota-derived metabolites modulating immunity (e.g. via STING–IFN axis) | Pan-cancer (multi-cohort anti-PD1/PD-L1 therapy) | Integration of fecal metagenomics and metabolomics in ICI-treated patients identified *five response-associated enterotypes*, each linked to specific bacteria and metabolites. Notably, the metabolite phenylacetylglutamine (PAGln) was **negatively** correlated with response: higher PAGln levels were seen in non-responders and experimental addition of PAGln attenuated PD-1 blockade efficacy in vivo[[6]](https://pubmed.ncbi.nlm.nih.gov/39909032/#:~:text=performance,This%20study%20sheds%20light%20on). These findings suggest that specific gut microbiome/metabolite signatures (especially PAGln) can predict ICI outcome across cancers. | [[6]](https://pubmed.ncbi.nlm.nih.gov/39909032/#:~:text=performance,This%20study%20sheds%20light%20on) |
| **IFN-stimulated neutrophils (LY6E^hi)** | Cellular (circulating immune cell subset) | IFN–STING pathway in neutrophils enhancing T cell activation (via IL-12) | Pan-cancer (various models and patient tumors) | A distinct subset of blood neutrophils characterized by high LY6E expression and an interferon-stimulated profile was found to predict ICI success. Patients with a higher frequency of these LY6E^hi neutrophils had better outcomes, and adoptive transfer of LY6E^hi neutrophils into poorly immunogenic tumors rendered them responsive to anti-PD1[[7]](https://www.nature.com/articles/s41577-024-00993-y?error=cookies_not_supported&code=9b0284c6-3c25-44f0-8a5d-53a3574e280f#:~:text=Immune%20checkpoint%20inhibitors%20,mouse%20models%20and%20in%20patients). Mechanistically, these neutrophils were induced by tumor STING–IFN signaling and directly promoted CD8+ T cell responses. | [[7]](https://www.nature.com/articles/s41577-024-00993-y?error=cookies_not_supported&code=9b0284c6-3c25-44f0-8a5d-53a3574e280f#:~:text=Immune%20checkpoint%20inhibitors%20,mouse%20models%20and%20in%20patients) |
| **Gasdermin D (GSDMD) expression** | Transcriptomic (pyroptosis executor) | Pyroptotic cell death shaping tumor-immune interactions | Pan-cancer (TCGA multiple tumor types) | In a pan-cancer study, GSDMD (which mediates inflammatory pyroptosis) was found overexpressed in many tumors and *positively* correlated with TMB, MSI, and immune checkpoint levels. Tumors with high GSDMD showed increased pro-inflammatory immune cell infiltration. This suggests that high GSDMD marks an inflamed TME and may predict responsiveness to ICI. The authors propose GSDMD as a potential predictive biomarker for immunotherapy[[8]](https://pubmed.ncbi.nlm.nih.gov/40491921/#:~:text=Results%3A%20%20GSDMD%20was%20found,responsive%20phenotype). | [[8]](https://pubmed.ncbi.nlm.nih.gov/40491921/#:~:text=Results%3A%20%20GSDMD%20was%20found,responsive%20phenotype) |

**Sources:** All findings are based on 2024–2025 peer-reviewed articles or conference abstracts. Citations in table refer to the specific studies or abstracts as listed. Each source link (e.g. “[[1]](https://s3.amazonaws.com/files.oncologymeetings.org/prod/s3fs-public/2024-05/AM24-Developmental-Therapeutics-Immunotherapy.pdf#:~:text=showed%20high%20CRTAM%20expression,significance%20was%20preserved%20in%20multivariate)”) corresponds to a published report or meeting abstract detailing the results.

[[1]](https://s3.amazonaws.com/files.oncologymeetings.org/prod/s3fs-public/2024-05/AM24-Developmental-Therapeutics-Immunotherapy.pdf#:~:text=showed%20high%20CRTAM%20expression,significance%20was%20preserved%20in%20multivariate) [[2]](https://s3.amazonaws.com/files.oncologymeetings.org/prod/s3fs-public/2024-05/AM24-Developmental-Therapeutics-Immunotherapy.pdf#:~:text=its%20enrichment%20in%20subsets%20of,over%20Chemo%20were%20lost%20in) 7.Developmental-Therapeutics24up 1..198

<https://s3.amazonaws.com/files.oncologymeetings.org/prod/s3fs-public/2024-05/AM24-Developmental-Therapeutics-Immunotherapy.pdf>

[[3]](https://www.nature.com/articles/s41698-024-00703-w?error=cookies_not_supported&code=da9116d5-b174-41ca-87f8-4dc79eebfa0c#:~:text=we%20utilized%20EcoTyper%20and%20publicly,machine%20learning%20models%20successfully%20predicted) Integrative analysis of pan-cancer single-cell data reveals a tumor ecosystem subtype predicting immunotherapy response | npj Precision Oncology

<https://www.nature.com/articles/s41698-024-00703-w?error=cookies_not_supported&code=da9116d5-b174-41ca-87f8-4dc79eebfa0c>

[[4]](https://www.researchgate.net/publication/392302319_Validation_of_ENLIGHT_an_AI_predictor_of_immune_checkpoint_blockade_ICB_response_and_resistance_across_the_treatment_span#:~:text=vs%200,6) Validation of ENLIGHT, an AI predictor of immune checkpoint blockade (ICB) response and resistance, across the treatment span. | Request PDF

<https://www.researchgate.net/publication/392302319_Validation_of_ENLIGHT_an_AI_predictor_of_immune_checkpoint_blockade_ICB_response_and_resistance_across_the_treatment_span>

[[5]](https://pmc.ncbi.nlm.nih.gov/articles/PMC11145176/#:~:text=demonstrate%20that%20early%20changes%20in,in%20patients%20treated%20with%20pembrolizumab) Early Changes in Tumor-Naive Cell-Free Methylomes and Fragmentomes Predict Outcomes in Pembrolizumab-Treated Solid Tumors - PMC

<https://pmc.ncbi.nlm.nih.gov/articles/PMC11145176/>

[[6]](https://pubmed.ncbi.nlm.nih.gov/39909032/#:~:text=performance,This%20study%20sheds%20light%20on) Interplay between gut microbial communities and metabolites modulates pan-cancer immunotherapy responses - PubMed

<https://pubmed.ncbi.nlm.nih.gov/39909032/>

[[7]](https://www.nature.com/articles/s41577-024-00993-y?error=cookies_not_supported&code=9b0284c6-3c25-44f0-8a5d-53a3574e280f#:~:text=Immune%20checkpoint%20inhibitors%20,mouse%20models%20and%20in%20patients) Blood neutrophil subset predicts the success of immune checkpoint inhibitors in cancer | Nature Reviews Immunology

<https://www.nature.com/articles/s41577-024-00993-y?error=cookies_not_supported&code=9b0284c6-3c25-44f0-8a5d-53a3574e280f>

[[8]](https://pubmed.ncbi.nlm.nih.gov/40491921/#:~:text=Results%3A%20%20GSDMD%20was%20found,responsive%20phenotype) GSDMD is a novel predictive biomarker for immunotherapy response: in the pan-cancer and single cell landscapes - PubMed

<https://pubmed.ncbi.nlm.nih.gov/40491921/>
